# 1,3-Bis(2-ethoxy­phen­yl)triazene methanol 0.33-solvate

**DOI:** 10.1107/S1600536809035338

**Published:** 2009-09-09

**Authors:** Mohammad Kazem Rofouei, Mohammad Reza Melardi, Yasaman Salemi, Jafar Attar Gharamaleki

**Affiliations:** a Faculty of Chemistry, Tarbiat Moallem University, Tehran, Iran; b Department of Chemistry, Islamic Azad University, Karaj Branch, Karaj, Iran; c Young Researchers Club, Islamic Azad University, North Tehran Branch, Tehran, Iran

## Abstract

There are three independent mol­ecules of 1,3-bis­(2-ethoxy­phen­yl)triazene and a mol­ecule of methanol in the asymmetric unit of the title compound, C_16_H_19_N_3_O_2_·0.33CH_3_OH. Two mol­ecules related by a non-crystallographic pseudo-twofold rotation axis are linked *via* distinct inter­molecular N—H⋯N hydrogen bonds, leading to the formation of a dimer with an *R*_2_^2^(8) graph set. The third mol­ecule is connected to the methanol mol­ecule by O—H⋯N and N—H⋯O hydrogen bonds. There are a number of weak C—H⋯π inter­actions, with H⋯π distances ranging from 2.74 to 2.89 Å between the C—H groups and the aromatic benzene rings.

## Related literature

For related structures, see: Rofouei *et al.* (2009 ▶); Melardi *et al.*(2008 ▶); Rofouei *et al.* (2006 ▶). For the structural properties and metal complexes of aryl triazenes, see: Meldola *et al.* (1888 ▶); Leman *et al.* (1993 ▶); Chen *et al.* (2002 ▶); Vrieze *et al.* (1987 ▶); Hematyar *et al.* (2008 ▶); Payehghadr *et al.* (2007 ▶). For hydrogen-bond patterns and graph sets, see: Grell *et al.* (2002 ▶).
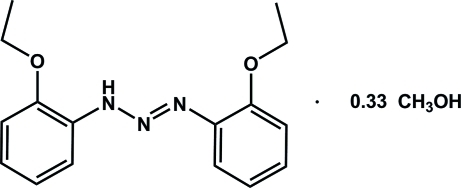


## Experimental

### 

#### Crystal data

C_16_H_19_N_3_O_2_·0.33CH_4_O
*M* *_r_* = 296.02
Triclinic, 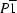

*a* = 12.146 (3) Å
*b* = 13.640 (3) Å
*c* = 16.117 (4) Å
α = 71.448 (5)°
β = 72.827 (4)°
γ = 81.151 (4)°
*V* = 2413.2 (10) Å^3^
*Z* = 6
Mo *K*α radiation
μ = 0.08 mm^−1^
*T* = 120 K
0.30 × 0.20 × 0.10 mm

#### Data collection

Bruker SMART 1000 CCD area-detector diffractometer
Absorption correction: multi-scan (*SADABS*; Bruker, 1998 ▶) *T*_min_ = 0.978, *T*_max_ = 0.992
21014 measured reflections
9421 independent reflections
4997 reflections with *I* > 2σ(*I*)
*R*_int_ = 0.048

#### Refinement

*R*[*F* ^2^ > 2σ(*F* ^2^)] = 0.060
*wR*(*F* ^2^) = 0.204
*S* = 1.00
9421 reflections
587 parameters
H-atom parameters constrained
Δρ_max_ = 0.31 e Å^−3^
Δρ_min_ = −0.30 e Å^−3^

### 

Data collection: *SMART* (Bruker, 1998 ▶); cell refinement: *SAINT-Plus* (Bruker, 1998 ▶); data reduction: *SAINT-Plus*; program(s) used to solve structure: *SHELXS97* (Sheldrick, 2008 ▶); program(s) used to refine structure: *SHELXL97* (Sheldrick, 2008 ▶); molecular graphics: *SHELXTL* (Sheldrick, 2008 ▶); software used to prepare material for publication: *SHELXTL*.

## Supplementary Material

Crystal structure: contains datablocks I, global. DOI: 10.1107/S1600536809035338/pv2190sup1.cif

Structure factors: contains datablocks I. DOI: 10.1107/S1600536809035338/pv2190Isup2.hkl

Additional supplementary materials:  crystallographic information; 3D view; checkCIF report

## Figures and Tables

**Table 1:** Hydrogen-bond geometry (Å, °)

*D*—H⋯*A*	*D*—H	H⋯*A*	*D*⋯*A*	*D*—H⋯*A*
N3—H3*N*⋯N4	0.88	2.20	3.024 (3)	156
N6—H6*N*⋯N1	0.88	2.20	3.033 (3)	158
N9—H9*N*⋯O7	0.88	2.19	2.920 (3)	140
O7—H7*O*⋯N7	0.88	2.15	2.839 (3)	134
C28—H28*A*⋯*Cg*1^i^	0.95	2.89	3.712 (3)	146
C36—H36*A*⋯*Cg*2^i^	0.95	2.74	3.549 (3)	144
C15—H15*A*⋯*Cg*3^ii^	0.99	2.76	3.463 (3)	128
C32—H32*C*⋯*Cg*3^i^	0.98	2.80	3.593 (3)	138
C40—H40*C*⋯*Cg*4^i^	0.98	2.84	3.632 (3)	138

Symmetry codes: (i) 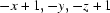; (ii) 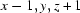. *Cg*1, *Cg*2, *Cg*3 and *Cg*4 are the centroids of the C1–C6, C9–C14, C33–C38 and C25–C30 rings, respectively.

**Fig. 1.**
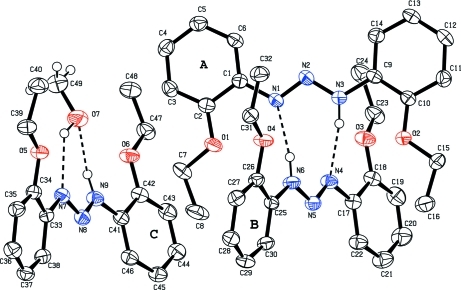
Molecular structure of the title compound, with thermal elliposids drawn at 50% probability level. Only hydrogen atoms involved in the hydrogen bonding are shown.

**Fig. 2.**
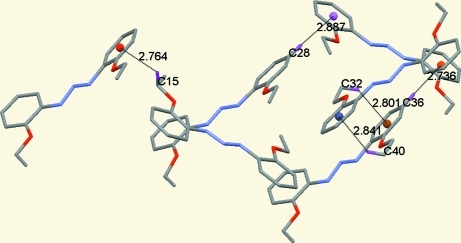
Weak C—H···π interactions between C–H groups with aromatic phenyl rings with with H···π distances ranging from 2.74 Å to 2.89 Å.

**Fig. 3.**
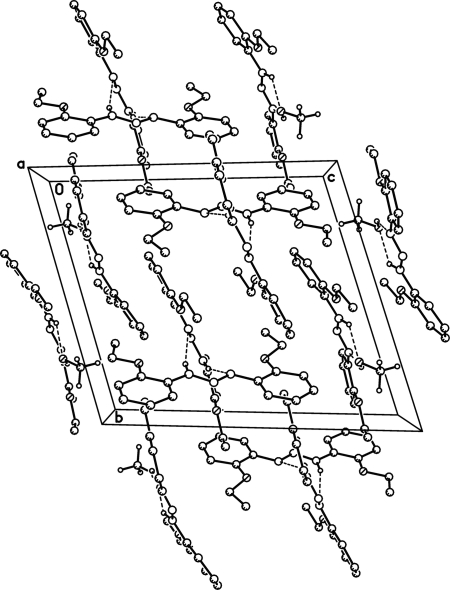
Unit cell packing diagram of the title compound. Hydrogen bonds are shown as dashed lines.

## supplementary crystallographic information

### Comment

Aryl triazenes have been studied over 130 years for their interesting structural, anticancer, and reactivity properties. The first extensive investigation of the coordination chemistry of a triazene derivative (1,3-diphenyltriazene) was carried out in 1887 by Meldola (Meldola *et al.*, 1888). In the intervening years, numerous transition metal triazenide compounds have been studied (Liman, *et al.*, 1993). Triazene compounds characterized by having a diazoamino group commonly adopt a *trans* configuration in the ground state (Chen *et al.*, 2002). The study of transition metal complexes containing 1,3-diaryltriazenide [RN═N—NR]^–^ ligands has increased greatly in the past few years, because of their potential reactivity in relation to their several coordination modes (Vrieze, *et al.*, 1987). We have recently reported the synthesis and characterization of three 1,3-bis derivatives of triazene (Melardi *et al.,* 2008; Rofouei *et al.,* 2006; Rofouei *et al.,* 2009).

The title structure contains three molecules of C_16_H_19_N_3_O_2_ and a molecule of CH_3_OH in an asymmetric unit (Fig. 1). It is similar to our recently published article, C_16_H_19_N_3_O_2_, [Rofouei, *et al.*, 2009] and only differs in one methanol molecule as solvent. All the three molecules A, B and C show *trans* stereo chemistry for the N═N double bond. The torsion angles C1—N1—N2—N3, C17—N4—N5—N6 and C33—N7—N8—N9 are -177.34 (17), 179.42 (16) and 177.30 (16)°, respectively. The N1—N2, N2—N3, N4—N5, N5—N6, N7—N8 and N8—N9 bond distances are 1.291 (2), 1.308 (3), 1.298 (2), 1.304 (3), 1.276 (3) and 1.328 (2) Å, respectively which are in good agreement with the reported data for N—N and N═N bond distances (Hematyar, *et al.*, 2008; Payehghadr, *et al.*, 2007; Melardi, *et al.*, 2008).

The molecule A is almost planar, but the other two molecules (B and C) are somewhat twisted with respect to the phenyl rings. Two interlocked molecules (A and B) are connected by two distinct classic N—H···N hydrogen bonds with D···A of 3.024 (3) and 3.033 (3) Å and are related by a non-crystallographic pseudo twofold rotation axis. The N—H···N hydrogen bonds lead to the formation of a dimer with an *R^2^_2_*(8) graph set geometry (Grell, *et al.*, 2002). The steric demand of the ethoxy groups in the *ortho* position prevents a co-planar arrangement of the two molecules in the dimer which instead consists of two interlocked molecules. The third molecule (C) is connected to a methanol molecule by two O7—H7O···N7 and N9—H9N···O7 hydrogen bonds forming a six membered ring with an *R^2^_2_*(6) graph set geometry (Grell, *et al.*, 2002). Hydrogen bond geometries are shown in Table 1.

Also, there are several interesting weak C—H···π interactions between CH groups with aromatic phenyl rings with H···π distances ranging from 2.74 Å to 2.89 Å (Fig. 2). The unit cell packing of the title compound is presented in Fig. 3.

### Experimental

A 100 ml flask was charged with 10 g of ice and 15 ml of water and then cooled to 273 K in an ice-bath. To this was added 10 mmol (1.37 g) of *o*–phenetidin and 13 mmol of hydrochloric acid (37%) followed by a solution containing NaNO_2_ 6 mmol (0.41 g) in 25 ml of water during a 15 min period. After mixing for 15 min a solution containing 180 mmol (14.76 g) of sodium acetate in 45 ml of water was added. After mixing for 45 min the brown product was filtered and dissolved in Et_2_O, and was crystallized at 263 K. Recrystallization from methanol afforded the title compound as an orange crystalline material.

### Refinement

The hydrogen atoms bonded to N and O were found from difference Fourier synthesis. All hydrogen atoms were included in the refinement at geometrically idealized positions in isotropic approximation in riding mode with distances: N/O–H = 0.88 Å, C–H = 0.95 (aryl), 0.98 (methyl), 0.99 (methylene) Å and *U*_iso_(H) equal to 1.5*U*_eq_(C) for methyl groups and 1.2*U*_eq_(N/O and methylene C).

### Crystal data

**Table:**

C_16_H_19_N_3_O_2_·0.33CH_4_O	*Z* = 6
*M**_r_* = 296.02	*F*(000) = 948
Triclinic, *P*1	*D*_x_ = 1.222 Mg m^−^^3^
Hall symbol: -P 1	Mo *K*α radiation, λ = 0.71073 Å
*a* = 12.146 (3) Å	Cell parameters from 2921 reflections
*b* = 13.640 (3) Å	θ = 2.4–24.6°
*c* = 16.117 (4) Å	µ = 0.08 mm^−^^1^
α = 71.448 (5)°	*T* = 120 K
β = 72.827 (4)°	Prism, orange
γ = 81.151 (4)°	0.30 × 0.20 × 0.10 mm
*V* = 2413.2 (10) Å^3^	

### Data collection

**Table:**

Bruker SMART 1000 CCD area-detector diffractometer	9421 independent reflections
Radiation source: fine-focus sealed tube	4997 reflections with *I* > 2σ(*I*)
graphite	*R*_int_ = 0.048
φ and ω scans	θ_max_ = 26.0°, θ_min_ = 1.8°
Absorption correction: multi-scan (*SADABS*; Bruker, 1998)	*h* = −14→14
*T*_min_ = 0.978, *T*_max_ = 0.992	*k* = −16→16
21014 measured reflections	*l* = −19→19

### Refinement

**Table:**

Refinement on *F*^2^	Primary atom site location: structure-invariant direct methods
Least-squares matrix: full	Secondary atom site location: difference Fourier map
*R*[*F*^2^ > 2σ(*F*^2^)] = 0.060	Hydrogen site location: difference Fourier map
*wR*(*F*^2^) = 0.204	H-atom parameters constrained
*S* = 1.00	*w* = 1/[σ^2^(*F*_o_^2^) + (0.07*P*)^2^ + 2*P*] where *P* = (*F*_o_^2^ + 2*F*_c_^2^)/3
9421 reflections	(Δ/σ)_max_ < 0.001
587 parameters	Δρ_max_ = 0.31 e Å^−^^3^
0 restraints	Δρ_min_ = −0.30 e Å^−^^3^

### Special details

**Table:**

Geometry. All e.s.d.'s (except the e.s.d. in the dihedral angle between two l.s. planes) are estimated using the full covariance matrix. The cell e.s.d.'s are taken into account individually in the estimation of e.s.d.'s in distances, angles and torsion angles; correlations between e.s.d.'s in cell parameters are only used when they are defined by crystal symmetry. An approximate (isotropic) treatment of cell e.s.d.'s is used for estimating e.s.d.'s involving l.s. planes.
Refinement. Refinement of *F*^2^ against ALL reflections. The weighted *R*-factor *wR* and goodness of fit *S* are based on *F*^2^, conventional *R*-factors *R* are based on *F*, with *F* set to zero for negative *F*^2^. The threshold expression of *F*^2^ > σ(*F*^2^) is used only for calculating *R*-factors(gt) *etc*. and is not relevant to the choice of reflections for refinement. *R*-factors based on *F*^2^ are statistically about twice as large as those based on *F*, and *R*- factors based on ALL data will be even larger.

### Fractional atomic coordinates and isotropic or equivalent isotropic displacement parameters (Å^2^)

**Table:**

	*x*	*y*	*z*	*U*_iso_*/*U*_eq_	
O1	0.31967 (13)	0.24889 (12)	0.40953 (10)	0.0360 (4)	
O2	0.00977 (12)	0.23580 (12)	0.82111 (10)	0.0334 (4)	
N1	0.12742 (15)	0.19444 (14)	0.53596 (12)	0.0312 (5)	
N2	0.03282 (15)	0.16622 (14)	0.59722 (12)	0.0289 (5)	
N3	0.02050 (15)	0.19607 (14)	0.66913 (12)	0.0314 (5)	
H3N	0.0725	0.2323	0.6722	0.038*	
C1	0.14480 (18)	0.16900 (16)	0.45427 (14)	0.0272 (5)	
C2	0.24629 (18)	0.20179 (16)	0.38631 (15)	0.0296 (6)	
C3	0.26635 (19)	0.18578 (17)	0.30162 (15)	0.0322 (6)	
H3A	0.3335	0.2099	0.2549	0.039*	
C4	0.18748 (19)	0.13438 (17)	0.28613 (15)	0.0344 (6)	
H4A	0.2017	0.1224	0.2288	0.041*	
C5	0.08808 (19)	0.10025 (18)	0.35339 (15)	0.0342 (6)	
H5A	0.0349	0.0646	0.3425	0.041*	
C6	0.06734 (18)	0.11877 (17)	0.43658 (15)	0.0303 (6)	
H6A	−0.0013	0.0966	0.4823	0.036*	
C7	0.41398 (19)	0.29920 (19)	0.33826 (16)	0.0372 (6)	
H7A	0.3845	0.3525	0.2903	0.045*	
H7B	0.4660	0.2480	0.3107	0.045*	
C8	0.4776 (2)	0.3482 (2)	0.37950 (18)	0.0545 (8)	
H8A	0.5427	0.3833	0.3325	0.082*	
H8B	0.5065	0.2947	0.4267	0.082*	
H8C	0.4253	0.3988	0.4064	0.082*	
C9	−0.07846 (17)	0.16844 (16)	0.74147 (14)	0.0265 (5)	
C10	−0.08259 (18)	0.18916 (16)	0.82198 (14)	0.0277 (5)	
C11	−0.17676 (19)	0.15921 (17)	0.89794 (15)	0.0322 (6)	
H11A	−0.1797	0.1719	0.9532	0.039*	
C12	−0.26562 (18)	0.11094 (17)	0.89177 (15)	0.0330 (6)	
H12A	−0.3296	0.0909	0.9430	0.040*	
C13	−0.26151 (18)	0.09184 (17)	0.81164 (15)	0.0322 (6)	
H13A	−0.3226	0.0588	0.8080	0.039*	
C14	−0.16852 (18)	0.12082 (17)	0.73660 (15)	0.0308 (6)	
H14A	−0.1664	0.1080	0.6815	0.037*	
C15	0.0103 (2)	0.25548 (18)	0.90324 (15)	0.0354 (6)	
H15A	0.0089	0.1895	0.9524	0.043*	
H15B	−0.0586	0.3004	0.9223	0.043*	
C16	0.1185 (2)	0.3083 (2)	0.88496 (17)	0.0480 (7)	
H16A	0.1210	0.3227	0.9402	0.072*	
H16B	0.1189	0.3735	0.8364	0.072*	
H16C	0.1861	0.2631	0.8664	0.072*	
O3	−0.03481 (12)	0.44284 (12)	0.63303 (11)	0.0377 (4)	
O4	0.33503 (12)	0.04227 (11)	0.62005 (11)	0.0346 (4)	
N4	0.17838 (15)	0.36547 (13)	0.63042 (12)	0.0297 (5)	
N5	0.28253 (15)	0.32739 (13)	0.63278 (12)	0.0286 (5)	
N6	0.30570 (15)	0.23841 (13)	0.61518 (12)	0.0306 (5)	
H6N	0.2531	0.2095	0.6047	0.037*	
C17	0.14665 (18)	0.46114 (16)	0.64961 (14)	0.0282 (5)	
C18	0.03205 (18)	0.50163 (17)	0.65099 (15)	0.0309 (6)	
C19	−0.0048 (2)	0.59563 (18)	0.66942 (16)	0.0387 (7)	
H19A	−0.0815	0.6237	0.6697	0.046*	
C20	0.0692 (2)	0.64865 (18)	0.68742 (18)	0.0435 (7)	
H20A	0.0426	0.7124	0.7011	0.052*	
C21	0.1819 (2)	0.60992 (18)	0.68580 (17)	0.0424 (7)	
H21A	0.2324	0.6470	0.6980	0.051*	
C22	0.2204 (2)	0.51652 (17)	0.66619 (15)	0.0334 (6)	
H22A	0.2980	0.4903	0.6641	0.040*	
C23	−0.13987 (19)	0.4905 (2)	0.60961 (16)	0.0402 (7)	
H23A	−0.1235	0.5531	0.5569	0.048*	
H23B	−0.1946	0.5113	0.6613	0.048*	
C24	−0.1903 (2)	0.4117 (2)	0.58680 (17)	0.0464 (7)	
H24A	−0.2624	0.4414	0.5704	0.070*	
H24B	−0.2061	0.3503	0.6395	0.070*	
H24C	−0.1354	0.3919	0.5356	0.070*	
C25	0.41736 (17)	0.18971 (16)	0.61329 (14)	0.0264 (5)	
C26	0.43230 (18)	0.08528 (16)	0.61546 (14)	0.0278 (5)	
C27	0.54021 (19)	0.03263 (18)	0.61434 (15)	0.0329 (6)	
H27A	0.5504	−0.0386	0.6169	0.039*	
C28	0.63264 (19)	0.08407 (18)	0.60955 (15)	0.0344 (6)	
H28A	0.7062	0.0479	0.6082	0.041*	
C29	0.61904 (19)	0.18722 (18)	0.60672 (15)	0.0349 (6)	
H29A	0.6829	0.2220	0.6034	0.042*	
C30	0.51106 (18)	0.24000 (17)	0.60880 (14)	0.0310 (6)	
H30A	0.5014	0.3110	0.6071	0.037*	
C31	0.34302 (19)	−0.06530 (16)	0.62645 (15)	0.0316 (6)	
H31A	0.3618	−0.1077	0.6836	0.038*	
H31B	0.4043	−0.0800	0.5749	0.038*	
C32	0.2277 (2)	−0.09031 (18)	0.62455 (17)	0.0388 (6)	
H32A	0.2299	−0.1638	0.6288	0.058*	
H32B	0.2101	−0.0479	0.5677	0.058*	
H32C	0.1678	−0.0755	0.6759	0.058*	
O5	0.70638 (12)	0.06592 (11)	0.13410 (10)	0.0317 (4)	
O6	0.36080 (12)	0.48301 (12)	0.11751 (11)	0.0362 (4)	
N7	0.68644 (15)	0.26576 (14)	0.11614 (12)	0.0294 (5)	
N8	0.67007 (15)	0.35282 (14)	0.13324 (12)	0.0293 (5)	
N9	0.57197 (15)	0.40407 (13)	0.11906 (12)	0.0307 (5)	
H9N	0.5298	0.3809	0.0942	0.037*	
C33	0.79371 (18)	0.21153 (16)	0.12693 (14)	0.0259 (5)	
C34	0.80398 (18)	0.10554 (16)	0.13416 (14)	0.0277 (5)	
C35	0.90725 (19)	0.04810 (17)	0.14109 (15)	0.0318 (6)	
H35A	0.9146	−0.0236	0.1455	0.038*	
C36	1.00003 (19)	0.09627 (18)	0.14159 (15)	0.0348 (6)	
H36A	1.0708	0.0569	0.1462	0.042*	
C37	0.99072 (19)	0.20022 (18)	0.13548 (15)	0.0333 (6)	
H37A	1.0546	0.2321	0.1361	0.040*	
C38	0.88745 (18)	0.25806 (17)	0.12842 (14)	0.0304 (6)	
H38A	0.8806	0.3296	0.1246	0.036*	
C39	0.7134 (2)	−0.04229 (17)	0.14138 (16)	0.0354 (6)	
H39A	0.7355	−0.0844	0.1973	0.043*	
H39B	0.7717	−0.0573	0.0885	0.043*	
C40	0.5947 (2)	−0.06709 (19)	0.14433 (17)	0.0426 (7)	
H40A	0.5955	−0.1407	0.1493	0.064*	
H40B	0.5740	−0.0249	0.0887	0.064*	
H40C	0.5380	−0.0518	0.1969	0.064*	
C41	0.53667 (18)	0.49534 (16)	0.14432 (14)	0.0282 (5)	
C42	0.42322 (18)	0.53686 (16)	0.14433 (15)	0.0297 (6)	
C43	0.3816 (2)	0.62422 (17)	0.17383 (16)	0.0364 (6)	
H43A	0.3051	0.6526	0.1740	0.044*	
C44	0.4523 (2)	0.67014 (17)	0.20317 (16)	0.0349 (6)	
H44A	0.4235	0.7292	0.2242	0.042*	
C45	0.56442 (19)	0.62991 (17)	0.20172 (15)	0.0336 (6)	
H45A	0.6123	0.6614	0.2217	0.040*	
C46	0.60702 (19)	0.54372 (16)	0.17116 (15)	0.0311 (6)	
H46A	0.6848	0.5177	0.1686	0.037*	
C47	0.24610 (19)	0.52416 (19)	0.11158 (17)	0.0386 (7)	
H47A	0.2488	0.5938	0.0671	0.046*	
H47B	0.1978	0.5302	0.1713	0.046*	
C48	0.1965 (2)	0.4504 (2)	0.08185 (18)	0.0491 (7)	
H48A	0.1181	0.4761	0.0771	0.074*	
H48B	0.1943	0.3818	0.1265	0.074*	
H48C	0.2450	0.4452	0.0227	0.074*	
O7	0.46759 (14)	0.23487 (14)	0.10190 (12)	0.0507 (5)	
H7O	0.5406	0.2123	0.0967	0.061*	
C49	0.4211 (2)	0.2023 (2)	0.04551 (18)	0.0534 (8)	
H49A	0.4724	0.2190	−0.0161	0.080*	
H49B	0.3448	0.2379	0.0443	0.080*	
H49C	0.4137	0.1273	0.0690	0.080*	

### Atomic displacement parameters (Å^2^)

**Table:**

	*U*^11^	*U*^22^	*U*^33^	*U*^12^	*U*^13^	*U*^23^
O1	0.0283 (8)	0.0449 (9)	0.0346 (8)	−0.0153 (7)	−0.0055 (7)	−0.0076 (7)
O2	0.0295 (8)	0.0445 (8)	0.0306 (7)	−0.0119 (7)	−0.0058 (6)	−0.0146 (6)
N1	0.0236 (9)	0.0422 (10)	0.0281 (9)	−0.0098 (8)	−0.0026 (7)	−0.0110 (8)
N2	0.0233 (9)	0.0320 (9)	0.0301 (9)	−0.0036 (8)	−0.0045 (7)	−0.0087 (8)
N3	0.0280 (9)	0.0401 (10)	0.0298 (9)	−0.0109 (8)	−0.0044 (7)	−0.0141 (8)
C1	0.0267 (10)	0.0265 (11)	0.0284 (10)	−0.0023 (9)	−0.0084 (9)	−0.0067 (9)
C2	0.0279 (11)	0.0273 (11)	0.0333 (11)	−0.0019 (9)	−0.0107 (9)	−0.0061 (9)
C3	0.0244 (11)	0.0377 (12)	0.0319 (11)	0.0000 (10)	−0.0070 (9)	−0.0079 (10)
C4	0.0347 (12)	0.0389 (12)	0.0346 (11)	0.0042 (10)	−0.0138 (9)	−0.0162 (10)
C5	0.0295 (11)	0.0355 (12)	0.0426 (12)	−0.0009 (10)	−0.0144 (10)	−0.0143 (10)
C6	0.0232 (10)	0.0338 (12)	0.0323 (11)	−0.0041 (9)	−0.0065 (9)	−0.0071 (9)
C7	0.0265 (11)	0.0391 (13)	0.0384 (13)	−0.0083 (10)	−0.0054 (10)	−0.0009 (10)
C8	0.0448 (14)	0.0673 (17)	0.0480 (15)	−0.0342 (13)	−0.0114 (12)	0.0014 (13)
C9	0.0215 (10)	0.0261 (11)	0.0304 (11)	−0.0017 (9)	−0.0076 (9)	−0.0056 (9)
C10	0.0229 (10)	0.0295 (11)	0.0297 (11)	−0.0021 (9)	−0.0068 (9)	−0.0073 (9)
C11	0.0321 (12)	0.0359 (12)	0.0271 (11)	−0.0017 (10)	−0.0067 (9)	−0.0082 (9)
C12	0.0205 (11)	0.0353 (12)	0.0357 (12)	−0.0034 (10)	−0.0016 (9)	−0.0048 (10)
C13	0.0210 (10)	0.0343 (12)	0.0384 (12)	−0.0047 (9)	−0.0077 (9)	−0.0053 (10)
C14	0.0276 (11)	0.0340 (12)	0.0328 (11)	−0.0011 (10)	−0.0111 (9)	−0.0100 (9)
C15	0.0391 (12)	0.0416 (12)	0.0296 (11)	−0.0075 (11)	−0.0096 (10)	−0.0130 (10)
C16	0.0536 (14)	0.0573 (15)	0.0396 (13)	−0.0226 (13)	−0.0123 (11)	−0.0138 (11)
O3	0.0254 (7)	0.0351 (8)	0.0549 (9)	0.0009 (7)	−0.0170 (7)	−0.0115 (7)
O4	0.0244 (7)	0.0296 (8)	0.0565 (9)	−0.0027 (6)	−0.0147 (7)	−0.0174 (7)
N4	0.0237 (9)	0.0265 (9)	0.0413 (10)	0.0005 (8)	−0.0109 (8)	−0.0121 (8)
N5	0.0264 (9)	0.0287 (9)	0.0331 (9)	−0.0029 (8)	−0.0091 (7)	−0.0105 (7)
N6	0.0246 (9)	0.0318 (9)	0.0428 (10)	−0.0024 (8)	−0.0128 (8)	−0.0169 (8)
C17	0.0285 (11)	0.0252 (11)	0.0303 (11)	−0.0015 (9)	−0.0096 (9)	−0.0057 (9)
C18	0.0276 (11)	0.0306 (11)	0.0337 (11)	−0.0051 (10)	−0.0091 (9)	−0.0056 (9)
C19	0.0310 (12)	0.0328 (13)	0.0471 (14)	0.0058 (10)	−0.0101 (10)	−0.0083 (11)
C20	0.0476 (14)	0.0275 (12)	0.0586 (15)	0.0040 (11)	−0.0169 (12)	−0.0173 (11)
C21	0.0454 (14)	0.0318 (12)	0.0563 (14)	−0.0017 (11)	−0.0190 (11)	−0.0167 (11)
C22	0.0309 (11)	0.0267 (11)	0.0450 (12)	−0.0008 (10)	−0.0140 (10)	−0.0103 (10)
C23	0.0271 (11)	0.0489 (15)	0.0403 (13)	−0.0016 (11)	−0.0130 (10)	−0.0036 (11)
C24	0.0311 (12)	0.0602 (16)	0.0468 (14)	−0.0024 (12)	−0.0165 (11)	−0.0086 (12)
C25	0.0202 (10)	0.0325 (11)	0.0276 (10)	−0.0022 (9)	−0.0069 (8)	−0.0095 (9)
C26	0.0255 (10)	0.0300 (11)	0.0315 (11)	−0.0014 (9)	−0.0111 (9)	−0.0105 (9)
C27	0.0314 (11)	0.0322 (12)	0.0385 (12)	0.0021 (10)	−0.0133 (9)	−0.0131 (9)
C28	0.0223 (10)	0.0445 (13)	0.0405 (12)	0.0034 (10)	−0.0130 (9)	−0.0159 (10)
C29	0.0239 (10)	0.0405 (13)	0.0440 (12)	−0.0068 (10)	−0.0135 (9)	−0.0109 (10)
C30	0.0289 (11)	0.0334 (11)	0.0368 (11)	−0.0043 (9)	−0.0128 (9)	−0.0138 (9)
C31	0.0330 (12)	0.0255 (11)	0.0373 (12)	−0.0024 (9)	−0.0071 (10)	−0.0123 (9)
C32	0.0396 (13)	0.0343 (12)	0.0462 (13)	−0.0080 (11)	−0.0119 (11)	−0.0136 (10)
O5	0.0277 (7)	0.0287 (8)	0.0415 (8)	−0.0052 (6)	−0.0109 (6)	−0.0107 (6)
O6	0.0265 (7)	0.0356 (8)	0.0526 (9)	−0.0013 (7)	−0.0171 (7)	−0.0152 (7)
N7	0.0251 (9)	0.0313 (9)	0.0330 (9)	−0.0027 (8)	−0.0065 (7)	−0.0116 (8)
N8	0.0263 (9)	0.0283 (9)	0.0324 (9)	−0.0016 (8)	−0.0075 (8)	−0.0078 (8)
N9	0.0238 (9)	0.0305 (10)	0.0391 (10)	0.0006 (8)	−0.0112 (8)	−0.0104 (8)
C33	0.0245 (10)	0.0299 (11)	0.0261 (10)	−0.0031 (9)	−0.0079 (8)	−0.0100 (8)
C34	0.0266 (11)	0.0287 (11)	0.0285 (10)	−0.0048 (9)	−0.0078 (9)	−0.0072 (9)
C35	0.0316 (11)	0.0284 (11)	0.0378 (11)	−0.0005 (10)	−0.0110 (9)	−0.0121 (9)
C36	0.0287 (11)	0.0391 (13)	0.0365 (12)	0.0021 (10)	−0.0144 (10)	−0.0076 (10)
C37	0.0262 (11)	0.0395 (13)	0.0367 (12)	−0.0067 (10)	−0.0104 (9)	−0.0101 (10)
C38	0.0301 (11)	0.0313 (11)	0.0323 (11)	−0.0039 (10)	−0.0096 (9)	−0.0105 (9)
C39	0.0400 (12)	0.0249 (11)	0.0419 (12)	−0.0087 (10)	−0.0118 (10)	−0.0062 (10)
C40	0.0463 (14)	0.0379 (13)	0.0453 (13)	−0.0162 (11)	−0.0119 (11)	−0.0081 (11)
C41	0.0272 (11)	0.0253 (11)	0.0295 (11)	−0.0038 (9)	−0.0065 (9)	−0.0044 (9)
C42	0.0275 (11)	0.0266 (11)	0.0334 (11)	−0.0026 (9)	−0.0103 (9)	−0.0039 (9)
C43	0.0289 (12)	0.0308 (12)	0.0464 (13)	0.0012 (10)	−0.0090 (10)	−0.0094 (10)
C44	0.0353 (12)	0.0250 (11)	0.0429 (13)	−0.0004 (10)	−0.0087 (10)	−0.0100 (10)
C45	0.0324 (12)	0.0269 (11)	0.0424 (12)	−0.0043 (10)	−0.0116 (10)	−0.0084 (10)
C46	0.0266 (11)	0.0276 (11)	0.0380 (12)	−0.0018 (9)	−0.0088 (9)	−0.0077 (9)
C47	0.0249 (11)	0.0469 (14)	0.0429 (13)	0.0008 (11)	−0.0116 (10)	−0.0106 (11)
C48	0.0328 (12)	0.0644 (17)	0.0580 (15)	−0.0062 (12)	−0.0187 (11)	−0.0210 (13)
O7	0.0327 (9)	0.0655 (11)	0.0647 (10)	−0.0042 (8)	−0.0145 (8)	−0.0315 (9)
C49	0.0505 (15)	0.0630 (17)	0.0545 (15)	−0.0103 (14)	−0.0170 (13)	−0.0214 (13)

### Geometric parameters (Å, °)

**Table:**

O1—C2	1.369 (3)	C24—H24B	0.9800
O1—C7	1.437 (3)	C24—H24C	0.9800
O2—C10	1.367 (3)	C25—C30	1.390 (3)
O2—C15	1.434 (3)	C25—C26	1.398 (3)
N1—N2	1.291 (2)	C26—C27	1.392 (3)
N1—C1	1.415 (3)	C27—C28	1.384 (3)
N2—N3	1.308 (3)	C27—H27A	0.9500
N3—C9	1.407 (3)	C28—C29	1.379 (3)
N3—H3N	0.8800	C28—H28A	0.9500
C1—C6	1.385 (3)	C29—C30	1.392 (3)
C1—C2	1.407 (3)	C29—H29A	0.9500
C2—C3	1.395 (3)	C30—H30A	0.9500
C3—C4	1.390 (3)	C31—C32	1.503 (3)
C3—H3A	0.9500	C31—H31A	0.9900
C4—C5	1.389 (3)	C31—H31B	0.9900
C4—H4A	0.9500	C32—H32A	0.9800
C5—C6	1.386 (3)	C32—H32B	0.9800
C5—H5A	0.9500	C32—H32C	0.9800
C6—H6A	0.9500	O5—C34	1.377 (3)
C7—C8	1.494 (4)	O5—C39	1.434 (3)
C7—H7A	0.9900	O6—C42	1.367 (3)
C7—H7B	0.9900	O6—C47	1.438 (3)
C8—H8A	0.9800	N7—N8	1.276 (3)
C8—H8B	0.9800	N7—C33	1.425 (3)
C8—H8C	0.9800	N8—N9	1.328 (2)
C9—C14	1.388 (3)	N9—C41	1.398 (3)
C9—C10	1.399 (3)	N9—H9N	0.8800
C10—C11	1.404 (3)	C33—C38	1.396 (3)
C11—C12	1.390 (3)	C33—C34	1.402 (3)
C11—H11A	0.9500	C34—C35	1.387 (3)
C12—C13	1.382 (3)	C35—C36	1.392 (3)
C12—H12A	0.9500	C35—H35A	0.9500
C13—C14	1.386 (3)	C36—C37	1.379 (3)
C13—H13A	0.9500	C36—H36A	0.9500
C14—H14A	0.9500	C37—C38	1.390 (3)
C15—C16	1.503 (3)	C37—H37A	0.9500
C15—H15A	0.9900	C38—H38A	0.9500
C15—H15B	0.9900	C39—C40	1.514 (3)
C16—H16A	0.9800	C39—H39A	0.9900
C16—H16B	0.9800	C39—H39B	0.9900
C16—H16C	0.9800	C40—H40A	0.9800
O3—C18	1.369 (3)	C40—H40B	0.9800
O3—C23	1.438 (3)	C40—H40C	0.9800
O4—C26	1.371 (3)	C41—C46	1.388 (3)
O4—C31	1.428 (3)	C41—C42	1.407 (3)
N4—N5	1.298 (2)	C42—C43	1.391 (3)
N4—C17	1.407 (3)	C43—C44	1.396 (4)
N5—N6	1.304 (3)	C43—H43A	0.9500
N6—C25	1.412 (3)	C44—C45	1.384 (3)
N6—H6N	0.8800	C44—H44A	0.9500
C17—C22	1.387 (3)	C45—C46	1.387 (3)
C17—C18	1.413 (3)	C45—H45A	0.9500
C18—C19	1.386 (3)	C46—H46A	0.9500
C19—C20	1.380 (4)	C47—C48	1.506 (4)
C19—H19A	0.9500	C47—H47A	0.9900
C20—C21	1.385 (4)	C47—H47B	0.9900
C20—H20A	0.9500	C48—H48A	0.9800
C21—C22	1.388 (3)	C48—H48B	0.9800
C21—H21A	0.9500	C48—H48C	0.9800
C22—H22A	0.9500	O7—C49	1.407 (3)
C23—C24	1.501 (4)	O7—H7O	0.8807
C23—H23A	0.9900	C49—H49A	0.9800
C23—H23B	0.9900	C49—H49B	0.9800
C24—H24A	0.9800	C49—H49C	0.9800
			
C2—O1—C7	117.58 (18)	C30—C25—C26	119.43 (19)
C10—O2—C15	117.73 (16)	C30—C25—N6	123.6 (2)
N2—N1—C1	115.95 (19)	C26—C25—N6	116.94 (19)
N1—N2—N3	112.46 (18)	O4—C26—C27	125.0 (2)
N2—N3—C9	117.88 (19)	O4—C26—C25	115.26 (18)
N2—N3—H3N	121.1	C27—C26—C25	119.7 (2)
C9—N3—H3N	121.1	C28—C27—C26	120.0 (2)
C6—C1—C2	119.2 (2)	C28—C27—H27A	120.0
C6—C1—N1	124.47 (18)	C26—C27—H27A	120.0
C2—C1—N1	116.3 (2)	C29—C28—C27	120.7 (2)
O1—C2—C3	124.33 (19)	C29—C28—H28A	119.7
O1—C2—C1	115.8 (2)	C27—C28—H28A	119.7
C3—C2—C1	119.9 (2)	C28—C29—C30	119.5 (2)
C4—C3—C2	119.6 (2)	C28—C29—H29A	120.2
C4—C3—H3A	120.2	C30—C29—H29A	120.2
C2—C3—H3A	120.2	C25—C30—C29	120.6 (2)
C5—C4—C3	120.8 (2)	C25—C30—H30A	119.7
C5—C4—H4A	119.6	C29—C30—H30A	119.7
C3—C4—H4A	119.6	O4—C31—C32	107.13 (18)
C6—C5—C4	119.3 (2)	O4—C31—H31A	110.3
C6—C5—H5A	120.4	C32—C31—H31A	110.3
C4—C5—H5A	120.4	O4—C31—H31B	110.3
C1—C6—C5	121.2 (2)	C32—C31—H31B	110.3
C1—C6—H6A	119.4	H31A—C31—H31B	108.5
C5—C6—H6A	119.4	C31—C32—H32A	109.5
O1—C7—C8	107.4 (2)	C31—C32—H32B	109.5
O1—C7—H7A	110.2	H32A—C32—H32B	109.5
C8—C7—H7A	110.2	C31—C32—H32C	109.5
O1—C7—H7B	110.2	H32A—C32—H32C	109.5
C8—C7—H7B	110.2	H32B—C32—H32C	109.5
H7A—C7—H7B	108.5	C34—O5—C39	117.07 (16)
C7—C8—H8A	109.5	C42—O6—C47	117.96 (18)
C7—C8—H8B	109.5	N8—N7—C33	113.74 (18)
H8A—C8—H8B	109.5	N7—N8—N9	112.79 (19)
C7—C8—H8C	109.5	N8—N9—C41	118.98 (19)
H8A—C8—H8C	109.5	N8—N9—H9N	120.5
H8B—C8—H8C	109.5	C41—N9—H9N	120.5
C14—C9—C10	119.84 (19)	C38—C33—C34	119.4 (2)
C14—C9—N3	123.3 (2)	C38—C33—N7	124.04 (19)
C10—C9—N3	116.9 (2)	C34—C33—N7	116.52 (19)
O2—C10—C9	116.08 (17)	O5—C34—C35	124.6 (2)
O2—C10—C11	124.3 (2)	O5—C34—C33	115.34 (18)
C9—C10—C11	119.6 (2)	C35—C34—C33	120.1 (2)
C12—C11—C10	119.6 (2)	C34—C35—C36	119.5 (2)
C12—C11—H11A	120.2	C34—C35—H35A	120.2
C10—C11—H11A	120.2	C36—C35—H35A	120.2
C13—C12—C11	120.4 (2)	C37—C36—C35	121.0 (2)
C13—C12—H12A	119.8	C37—C36—H36A	119.5
C11—C12—H12A	119.8	C35—C36—H36A	119.5
C12—C13—C14	120.2 (2)	C36—C37—C38	119.7 (2)
C12—C13—H13A	119.9	C36—C37—H37A	120.2
C14—C13—H13A	119.9	C38—C37—H37A	120.2
C13—C14—C9	120.4 (2)	C37—C38—C33	120.3 (2)
C13—C14—H14A	119.8	C37—C38—H38A	119.8
C9—C14—H14A	119.8	C33—C38—H38A	119.8
O2—C15—C16	107.77 (18)	O5—C39—C40	106.65 (18)
O2—C15—H15A	110.2	O5—C39—H39A	110.4
C16—C15—H15A	110.2	C40—C39—H39A	110.4
O2—C15—H15B	110.2	O5—C39—H39B	110.4
C16—C15—H15B	110.2	C40—C39—H39B	110.4
H15A—C15—H15B	108.5	H39A—C39—H39B	108.6
C15—C16—H16A	109.5	C39—C40—H40A	109.5
C15—C16—H16B	109.5	C39—C40—H40B	109.5
H16A—C16—H16B	109.5	H40A—C40—H40B	109.5
C15—C16—H16C	109.5	C39—C40—H40C	109.5
H16A—C16—H16C	109.5	H40A—C40—H40C	109.5
H16B—C16—H16C	109.5	H40B—C40—H40C	109.5
C18—O3—C23	118.40 (18)	C46—C41—N9	123.0 (2)
C26—O4—C31	118.17 (16)	C46—C41—C42	119.7 (2)
N5—N4—C17	115.73 (19)	N9—C41—C42	117.3 (2)
N4—N5—N6	112.45 (18)	O6—C42—C43	125.2 (2)
N5—N6—C25	118.21 (19)	O6—C42—C41	115.1 (2)
N5—N6—H6N	120.9	C43—C42—C41	119.6 (2)
C25—N6—H6N	120.9	C42—C43—C44	120.0 (2)
C22—C17—N4	124.1 (2)	C42—C43—H43A	120.0
C22—C17—C18	119.4 (2)	C44—C43—H43A	120.0
N4—C17—C18	116.5 (2)	C45—C44—C43	120.2 (2)
O3—C18—C19	124.8 (2)	C45—C44—H44A	119.9
O3—C18—C17	115.8 (2)	C43—C44—H44A	119.9
C19—C18—C17	119.4 (2)	C44—C45—C46	120.2 (2)
C20—C19—C18	120.3 (2)	C44—C45—H45A	119.9
C20—C19—H19A	119.8	C46—C45—H45A	119.9
C18—C19—H19A	119.8	C45—C46—C41	120.3 (2)
C19—C20—C21	120.7 (2)	C45—C46—H46A	119.8
C19—C20—H20A	119.6	C41—C46—H46A	119.8
C21—C20—H20A	119.6	O6—C47—C48	107.2 (2)
C20—C21—C22	119.6 (2)	O6—C47—H47A	110.3
C20—C21—H21A	120.2	C48—C47—H47A	110.3
C22—C21—H21A	120.2	O6—C47—H47B	110.3
C17—C22—C21	120.6 (2)	C48—C47—H47B	110.3
C17—C22—H22A	119.7	H47A—C47—H47B	108.5
C21—C22—H22A	119.7	C47—C48—H48A	109.5
O3—C23—C24	107.0 (2)	C47—C48—H48B	109.5
O3—C23—H23A	110.3	H48A—C48—H48B	109.5
C24—C23—H23A	110.3	C47—C48—H48C	109.5
O3—C23—H23B	110.3	H48A—C48—H48C	109.5
C24—C23—H23B	110.3	H48B—C48—H48C	109.5
H23A—C23—H23B	108.6	C49—O7—H7O	111.0
C23—C24—H24A	109.5	O7—C49—H49A	109.5
C23—C24—H24B	109.5	O7—C49—H49B	109.5
H24A—C24—H24B	109.5	H49A—C49—H49B	109.5
C23—C24—H24C	109.5	O7—C49—H49C	109.5
H24A—C24—H24C	109.5	H49A—C49—H49C	109.5
H24B—C24—H24C	109.5	H49B—C49—H49C	109.5
			
C1—N1—N2—N3	−177.34 (17)	C18—O3—C23—C24	175.79 (18)
N1—N2—N3—C9	−178.95 (17)	N5—N6—C25—C30	−15.0 (3)
N2—N1—C1—C6	1.2 (3)	N5—N6—C25—C26	165.52 (18)
N2—N1—C1—C2	178.45 (18)	C31—O4—C26—C27	1.6 (3)
C7—O1—C2—C3	10.1 (3)	C31—O4—C26—C25	−177.33 (18)
C7—O1—C2—C1	−169.62 (18)	C30—C25—C26—O4	179.89 (19)
C6—C1—C2—O1	−178.68 (19)	N6—C25—C26—O4	−0.6 (3)
N1—C1—C2—O1	3.9 (3)	C30—C25—C26—C27	0.9 (3)
C6—C1—C2—C3	1.6 (3)	N6—C25—C26—C27	−179.60 (19)
N1—C1—C2—C3	−175.85 (19)	O4—C26—C27—C28	180.0 (2)
O1—C2—C3—C4	178.2 (2)	C25—C26—C27—C28	−1.1 (3)
C1—C2—C3—C4	−2.1 (3)	C26—C27—C28—C29	0.6 (3)
C2—C3—C4—C5	1.0 (3)	C27—C28—C29—C30	0.1 (3)
C3—C4—C5—C6	0.6 (3)	C26—C25—C30—C29	−0.2 (3)
C2—C1—C6—C5	0.1 (3)	N6—C25—C30—C29	−179.7 (2)
N1—C1—C6—C5	177.3 (2)	C28—C29—C30—C25	−0.3 (3)
C4—C5—C6—C1	−1.1 (3)	C26—O4—C31—C32	−176.52 (18)
C2—O1—C7—C8	177.48 (19)	C33—N7—N8—N9	177.30 (16)
N2—N3—C9—C14	−6.8 (3)	N7—N8—N9—C41	173.13 (17)
N2—N3—C9—C10	171.86 (18)	N8—N7—C33—C38	−18.7 (3)
C15—O2—C10—C9	−178.17 (18)	N8—N7—C33—C34	162.85 (18)
C15—O2—C10—C11	−0.1 (3)	C39—O5—C34—C35	−0.3 (3)
C14—C9—C10—O2	179.74 (18)	C39—O5—C34—C33	179.99 (18)
N3—C9—C10—O2	1.0 (3)	C38—C33—C34—O5	178.60 (18)
C14—C9—C10—C11	1.5 (3)	N7—C33—C34—O5	−2.9 (3)
N3—C9—C10—C11	−177.20 (19)	C38—C33—C34—C35	−1.1 (3)
O2—C10—C11—C12	−179.1 (2)	N7—C33—C34—C35	177.37 (19)
C9—C10—C11—C12	−1.1 (3)	O5—C34—C35—C36	−179.2 (2)
C10—C11—C12—C13	0.3 (3)	C33—C34—C35—C36	0.5 (3)
C11—C12—C13—C14	0.0 (3)	C34—C35—C36—C37	0.1 (3)
C12—C13—C14—C9	0.5 (3)	C35—C36—C37—C38	−0.2 (3)
C10—C9—C14—C13	−1.3 (3)	C36—C37—C38—C33	−0.4 (3)
N3—C9—C14—C13	177.4 (2)	C34—C33—C38—C37	1.1 (3)
C10—O2—C15—C16	−179.05 (19)	N7—C33—C38—C37	−177.32 (19)
C17—N4—N5—N6	179.42 (16)	C34—O5—C39—C40	177.32 (18)
N4—N5—N6—C25	178.42 (17)	N8—N9—C41—C46	9.9 (3)
N5—N4—C17—C22	3.0 (3)	N8—N9—C41—C42	−168.33 (18)
N5—N4—C17—C18	−177.49 (18)	C47—O6—C42—C43	5.7 (3)
C23—O3—C18—C19	16.0 (3)	C47—O6—C42—C41	−176.92 (18)
C23—O3—C18—C17	−163.72 (18)	C46—C41—C42—O6	−179.48 (18)
C22—C17—C18—O3	179.33 (19)	N9—C41—C42—O6	−1.2 (3)
N4—C17—C18—O3	−0.2 (3)	C46—C41—C42—C43	−1.9 (3)
C22—C17—C18—C19	−0.4 (3)	N9—C41—C42—C43	176.37 (19)
N4—C17—C18—C19	−179.97 (19)	O6—C42—C43—C44	177.3 (2)
O3—C18—C19—C20	179.5 (2)	C41—C42—C43—C44	0.0 (3)
C17—C18—C19—C20	−0.8 (3)	C42—C43—C44—C45	1.0 (3)
C18—C19—C20—C21	1.2 (4)	C43—C44—C45—C46	0.0 (3)
C19—C20—C21—C22	−0.3 (4)	C44—C45—C46—C41	−1.9 (3)
N4—C17—C22—C21	−179.2 (2)	N9—C41—C46—C45	−175.31 (19)
C18—C17—C22—C21	1.3 (3)	C42—C41—C46—C45	2.9 (3)
C20—C21—C22—C17	−1.0 (4)	C42—O6—C47—C48	−179.43 (18)

### Hydrogen-bond geometry (Å, °)

**Table:**

*D*—H···*A*	*D*—H	H···*A*	*D*···*A*	*D*—H···*A*
N3—H3N···N4	0.88	2.20	3.024 (3)	156
N6—H6N···N1	0.88	2.20	3.033 (3)	158
N9—H9N···O7	0.88	2.19	2.920 (3)	140
O7—H7O···N7	0.88	2.15	2.839 (3)	134
C28—H28A···Cg1^i^	0.95	2.89	3.712 (3)	146
C36—H36A···Cg2^i^	0.95	2.74	3.549 (3)	144
C15—H15A···Cg3^ii^	0.99	2.76	3.463 (3)	128
C32—H32C···Cg3^i^	0.98	2.80	3.593 (3)	138
C40—H40C···Cg4^i^	0.98	2.84	3.632 (3)	138

Symmetry codes:  (i) −*x*+1, −*y*, −*z*+1; (ii) *x*−1, *y*, *z*+1.
